# Primary care models for treating opioid use disorders: What actually works? A systematic review

**DOI:** 10.1371/journal.pone.0186315

**Published:** 2017-10-17

**Authors:** Pooja Lagisetty, Katarzyna Klasa, Christopher Bush, Michele Heisler, Vineet Chopra, Amy Bohnert

**Affiliations:** 1 Division of General Internal Medicine, University of Michigan School of Medicine, Ann Arbor, Michigan, United States of America; 2 VA Center for Clinical Management Research, VA Ann Arbor Healthcare System, Ann Arbor, Michigan, United States of America; 3 Institute for Health Policy and Innovation, University of Michigan, Ann Arbor, Michigan, United States of America; 4 University of Michigan School of Public Health, Ann Arbor, Michigan, United States of America; 5 Department of Population Health Sciences, School of Medicine, Duke University, Durham, North Carolina, United States of America; 6 Division of Psychiatry, University of Michigan School of Medicine, Ann Arbor, Michigan, United States of America; Stanford University School of Medicine, UNITED STATES

## Abstract

**Background:**

Primary care-based models for Medication-Assisted Treatment (MAT) have been shown to reduce mortality for Opioid Use Disorder (OUD) and have equivalent efficacy to MAT in specialty substance treatment facilities.

**Objective:**

The objective of this study is to systematically analyze current evidence-based, primary care OUD MAT interventions and identify program structures and processes associated with improved patient outcomes in order to guide future policy and implementation in primary care settings.

**Data sources:**

PubMed, EMBASE, CINAHL, and PsychInfo.

**Methods:**

We included randomized controlled or quasi experimental trials and observational studies evaluating OUD treatment in primary care settings treating adult patient populations and assessed structural domains using an established systems engineering framework.

**Results:**

We included 35 interventions (10 RCTs and 25 quasi-experimental interventions) that all tested MAT, buprenorphine or methadone, in primary care settings across 8 countries. Most included interventions used joint multi-disciplinary (specialty addiction services combined with primary care) and coordinated care by physician and non-physician provider delivery models to provide MAT. Despite large variability in reported patient outcomes, processes, and tasks/tools used, similar key design factors arose among successful programs including integrated clinical teams with support staff who were often advanced practice clinicians (nurses and pharmacists) as clinical care managers, incorporating patient “agreements,” and using home inductions to make treatment more convenient for patients and providers.

**Conclusions:**

The findings suggest that multidisciplinary and coordinated care delivery models are an effective strategy to implement OUD treatment and increase MAT access in primary care, but research directly comparing specific structures and processes of care models is still needed.

## Introduction

Recent spikes in opioid-related overdoses have led experts to advocate for the creation of primary care-based treatment models to expand access to treatment for Opioid Use Disorders (OUD). [1, 2] OUD is categorized by individuals exhibiting signs and symptoms of compulsive behavior related to the self-administration of opioid substances [3]without a legitimate medical cause or in doses excess of what is clinically [2] necessary.[2] Internationally, of an estimated 48.9 million opioid/opiate users, 187,100 experience drug-related deaths annually.[4] In the US alone, about 2.5 million citizens have OUD, with an estimated 60,000 deaths due to drug overdoses [3] occurring annually.[5] A paucity of specialized substance treatment facilities and rising demand for OUD treatment presents primary care-based models the opportunity to increase access to treatment.

Over the past 15 years, health systems have developed and tested models to incorporate the use of medication-assisted treatment (MAT), also referred to as opioid-assisted treatment, into primary care settings. MAT uses pharmacological treatments such as buprenorphine and methadone coupled with psychosocial care to treat patients with OUD. [6] Primary care-based models for MAT appear to have roughly equivalent efficacy and outcomes to specialty substance treatment facilities in certain populations with the added advantage of managing, and potentially improving, comorbidity outcomes[7–12]. A recent scoping review has only looked at U.S. models, but no systematic, rigorous international comparisons with a focus on implementation structures and processes of OUD MAT in primary care settings exist to date [13]. No studies have attempted to synthesize the core implementation structures of these interventions, and as a result, little is known about the components included in effective models in primary care settings. This gap in the literature demonstrates the need to identify which components of primary care models for OUD treatment have shown success in implementation and acceptance by patients.

This systematic review aims to evaluate the literature on interventions for treating OUD in primary care settings using an established systems design framework: Systems Engineering Initiative for Patient Safety (SEIPS) 2.0 [14]. We use this framework to answer the questions: what structural characteristics and implementation components are described in existing primary care models for treating OUD, and how can we improve upon them in the future? Specifically, we aim to: (1) identify thematic components of primary care OUD MAT models that are accepted by patients and physicians and associated with improved health outcomes (2) use those findings to guide future policy and provide recommendations on design features of delivery models found to be effective in the primary care setting.

## Methods

### Data sources and searches

We followed the Preferred Reporting Items for Systematic Reviews and Meta-Analyses (PRISMA) recommendations in conducting this systematic review (PROSPERO 2016: CRD42016033762) [15]. With the assistance of a medical research librarian (MC), we performed serial literature searches for English language articles. MEDLINE via PubMed, CINAHL, EMBASE, and PsychInfo were searched for studies published prior to August 1, 2016 using Medical Subject Headings (MeSH) and keywords based on primary care settings and treatment of OUDs (S1 Table). All human studies published in full-text were eligible for inclusion, and no publication date or status restrictions were placed. Additional studies of interest were identified by hand searches of bibliographies. Authors were contacted by email if further clarification was needed.

### Study selection

Two authors (KK and CB) independently screened titles and abstracts for eligibility. Given the complexity of designing and evaluating care models [16], we included both experimental (RCTs) and observational studies (cohort, case-control, cross-sectional) if they met inclusion criteria. Articles were included if the intervention: (1) evaluated a primary care-based health delivery model where primary care was defined as care delivered in a general practice setting (i.e. private practice, academic primary care clinic) by a general medical internist and/or family medicine physician only, (2) targeted adults (18 years or older) with OUD defined as patients engaged in care to treat their opioid addiction, (3) evaluated patient-level outcomes (e.g. patient retention, urine toxicology screens, satisfaction, effect on health screening for comorbidities, etc.), and (4) evaluated the care model using qualitative or quantitative methods. Studies that did not include a description of the care delivery model evaluated (i.e. only discussed physician perceptions of OUD or drug dosage efficacy studies), focused exclusively on comparing intervention settings (e.g. specialty care versus primary care settings) without a detailed description of the primary care intervention/program design, and concentrated on specialty-based primary care (e.g. HIV care) outside of a primary care physician (PCP) led primary care practice were excluded (S2 Table). In the event of a disagreement in exclusion or inclusion between the two reviewers, a third reviewer (PL) resolved the discrepancy.

### Data extraction and quality assessment

Two authors (KK and CB) used a standardized form adapted from the Cochrane Collaboration [17, 18] to extract data from the included studies, independently and in duplicate. The following data was extracted for all studies: location, study design, intervention design and duration, care model structures and processes, classification, delivery staff, sample size, patient population, and primary/secondary outcomes as stated by the authors of each study.

Two authors (KK and CB) independently assessed risk of bias via the validated Downs and Black tool which utilizes the following elements to assess risk of bias in both experimental and observational studies: quality of reporting, internal validity of the study and its power, and external validity and confounding [19]. The tool evaluates each of these elements using 27 questions, allowing each article to receive a sum score of up to 32 points. For the purposes of this study, the last question assessing statistical power was interpreted as a dichotomous outcome: 0 for insufficient/no power calculation and 1 for studies that provided evidence of power calculation or reference to statistical power. From this alteration, 28 was the highest score possible. As previously reported [20] [21] the following were the final score ranges: excellent (26–28); good (20–25); fair (15–19); and poor (⩽ 14). Any discrepancies or disagreements in data review, extraction, or assessment of risk of bias were resolved by a third author (PL).

### Data synthesis and analysis

Given substantial clinical heterogeneity in patient outcomes reported (i.e. retention, relapse rates, comorbidity management, satisfaction, etc.) and variability in the drug treatments and dosages used in the models (e.g. methadone versus buprenorphine) within the included studies, formal meta-analyses were not performed.

## Results

### Identification of studies

**Fig 1:**
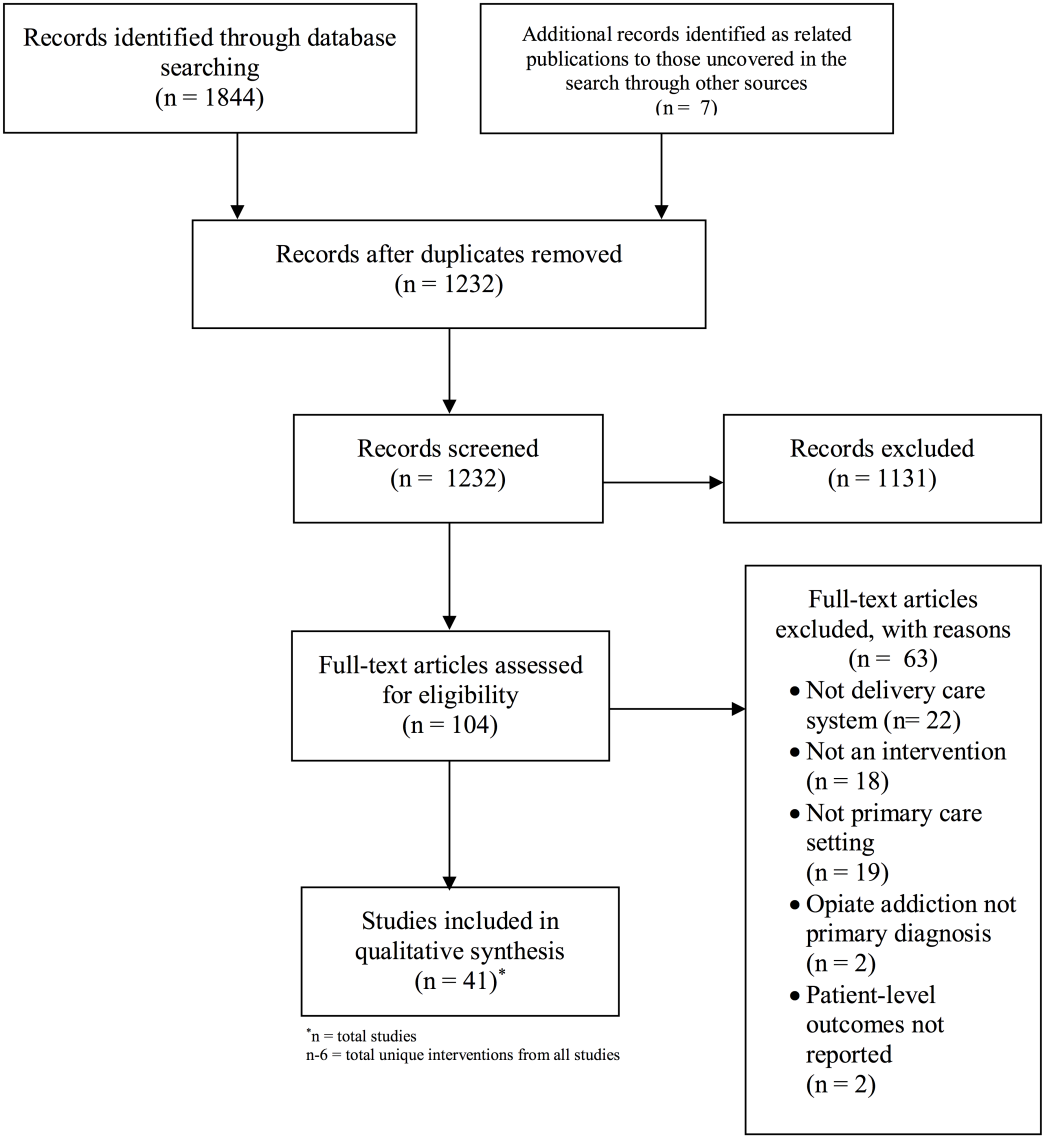
PRISMA flow diagram.

The database search retrieved a total of 1,844 articles and 7 articles identified as related publications to those uncovered in the search through other sources. Initial screening eliminated 1,131 articles at the title and abstract level for not fitting the inclusion criteria. Following full review of each of the remaining 104 articles, 63 articles were eliminated because they did not meet inclusion criteria, leading to 41 included publications (Fig 1). Reasons for exclusion of full-text studies included not a delivery care system, not an intervention, not a primary care setting, opiate addiction not primary care diagnosis, and unreported patient-level outcomes. The qualitative synthesis included 41 publications that described a total of 35 unique interventions. Two included models each had >1 publications that reported different outcomes from the same study (implementation outcomes and patient outcomes), which required inclusion of multiple publications for the same study to best evaluate the model’s efficacy. Of these unique interventions (n = 35), there were 10 randomized controlled trials and 25 quasi-experimental or observational studies.

### Study findings

We present the general study characteristics including settings and outcomes within the appropriate SEIPS domains to concisely summarize the findings without duplication of reporting. In addition, we present case-by-case examples of barriers and facilitators for the interventions using the SEIPS organizational framework (Table 1).

**Table 1:** SEIPS and study characteristics.

Author	Study Design	Environment	Organization	Person	Tasks	Process	Tech. & Tools	Patient Outcomes^a^	Provider Outcomes	Quality Rating
Alford et al (2008)	QE Comparison group (homeless clinic vs primary care) Total n = 85	Homeless Clinic vs. traditional PCP setting Boston, MA, USA Urban Academic PCC	Coordinated Care Model between PC physician and Nurse Care Manager Model(s): Coordinated Care **Duration**: 12 mo **Medication**: Buprenorphine	**PCP**: general internists **NCM**: Nurse Care Manager	**PCP**: overviewed prescribing and confirmed results of physical and oversaw NCM **NCM**: conducted home phone calls, initial assessment, tracked patients, on-call 24/7	*Staff*: NCM completed initial assessment with PC confirming physical assessment NCM available 24 hours a day via cellphone for patients Scheduled induction Patients went through 4 step process: 1) eligibility verification 2) medication induction 3) medication stabilization 4) treatment maintenance *Patient*: Encouraged to engage in self-help groups/therapy (recommended and tracked), but no individual counseling explicitly given; "Intensified treatment" (substance abuse counseling) was provided to patients with ongoing opioid, other drugs, or alcohol use	Cell phone with NCM 24/7 Patient contract	Retention: 55% in Homeless vs. 61% in Housed at 12 mo. Treatment failure, drug use, and utilization of substance abuse treatment services were examined	Not discussed	18 Fair
Alford et al (2011)	QE No comparison group Total N = 408	Boston Medical Center Urban academic primary care center Boston, MA, USA Urban Academic PCC	Multi-disciplinary Care Model with PCP, Nurse Director, and NCM with 1 program coordinator (medical assistant) Model(s): *Coordinated Care*, *Multi-disciplinary Care* **Duration**: 2 mo **Medication**: Buprenorphine	**PCP**: general internist **NCM**: Nurse care manager with 1-day training in BP **RN**: Nurse program director **Program Coordinator**: Medical assistant	**PCP**: generalists with part-time clinical practices, reviewed and supplemented the NCM assessments (including laboratory results), performed physical examinations, prescribed buprenorphine, and followed patients at least every 6 months (more if needed) **NCM**: assessed qualification for OBOT assessment, education, obtained informed consent, developed treatment plans, oversaw medication management (direct supervision of BP), referrals, monitored for treatment adherence, and communicated with PCP, addiction counselors, and pharmacists **Nurse Program Director**: oversaw the NCM **Program Coordinator**: medical assistant trained to collect standardized intake information for individuals requesting OBOT	*Staff*: The treatment model included 3 stages: (1) NCM and physician assessment (appropriateness for OBOT and intake evaluations), (2) NCM-supervised induction and stabilization (buprenorphine dose adjustments on days 1–7) (3) maintenance (buprenorphine treatment with monitoring for illicit drug use and weekly counseling) or discharge (voluntary or involuntary) *Patient*: Encouraged patients to seek outside individual or group counseling, but NCM provided education and support; Required to follow prescribing guidelines, attend follow-up, and provide urine samples	Patient contract	Retention: 51.3% at 12 mo. At 12 mo, 91.1% of patients remaining in treatment had negative urine drug tests	Open communication between the NCM and addiction counselors improved patients’ ability to comply with addiction care	18 Fair
Carrieri et al (2014)	RCT Comparison group (primary care vs specialized care) Total n = 221	BP is accessible in PC as of 2014 in France; only SC provides methadone SC can transfer patients to PC after methadone stabilization takes place (~14 days, randomized in study) North, North-Eastern, South-Western and South-Eastern France Urban PCC & SCC	Multi-disciplinary Model between specialty care and primary care with a multidisciplinary team including pharmacists Model(s): *Coordinated Care*, *Multi-disciplinary Care* **Duration**: 12 mo, 3 and 6 mo follow up **Medication**: Methadone	**PCP, SCP**: some had training treating OUD **Pharmacist & staff**	**PCPs** and **SCP**: supervised methadone induction for 2+ wks **Pharmacists**: supervised methadone induction for 2+ wks Pharmacists and physicians involved in the trial had to report overdoses, signs of intoxication and lost-to-follow-up patients to the center of methodology and management	*Staff*: All PCP and SCP had 1-day training for standardized methadone induction trial guidelines and procedures The PCP was responsible for dosing and supervision of induction after randomization occurred Starting dose: 30-40mg, with 10mg increases every 2–4 days until patient is stabilized The "intervention" included PCP or SCP doing supervised induction for at least 14 days for patient, then thereafter supervision only required in patients with overdose risk CATI after each visit lasting max 30 min Followed up with medical visits and phone interviews *Patient*: pre-enrollment medical questionnaire questionnaire at each scheduled visit (enrollment, 3, 6 and 12 mo) short self-administered questionnaire at all scheduled visits urine rapid tests when available	Phone calls to patients CATI = Computer Assisted Telephone Interview	Retention: Total sample: 73% at 12 mo.; 73% in PC and 50% in SC Self reported abstinence from street opioids 55% abstinent in PC, 33% in SC at 12 mo Higher satisfaction rates reported in PC-induced patients vs. SC (higher satisfaction with the explanations provided by PCP)* Engagement in treatment significantly lower in SC than in PC Early discontinuation rates significantly higher in the PC	Not discussed	23 Good
Colameco et al (2005)	QE No comparison group Total n = 35	Philadelphia, PA Urban Family Practice Center Referrals to the PCP were given mostly by trained addictions counselors already working with the population	Multi-disciplinary Model in which addiction counselor referred patients to PCP who then communicated with other treatment providers, family members, and patient pharmacies Model(s): *Coordinated Care*, *Multi-disciplinary Care* **Duration**: 12 mo **Medication**: Buprenorphine	**PCP**: certified addiction specialist **SCP** **Trained addiction counselors**	**PCP**: interviewed the patient over the phone prior to study enrollment, oversaw prescriptions and monitoring **Trained addiction counselors**: referred patients to PCP	*Staff*: PCP interviewed potential patients for 1 hour, discussed BP induction, and provided information packet Initial visit included: potential risks and benefits of treatment when compared to alternatives, admission criteria, and program requirements for ongoing treatment *Patient*: All patients required to participate in "group” counseling of choice (Narcotics Anonymous, faith-based programs, or group therapy at addiction treatment center) and individual MD counseling at center Patients had to return for monitoring 1x per month minimum: 30-minute assessments of treatment progress, and included clinical evaluation, drug testing, and communication with treatment providers, pharmacist, and family members	Phone calls to patients Patient contract	Retention: 62.9% at 12 mo. Patients with prescription coupon had higher probability remaining in treatment vs. those without*	Barriers to physician interest included the psychiatric comorbidities, a lack of PC addiction fellowship, and a lack of specific education with regards to this population	17 Fair
Cunningham et al (2008)	Observational Cohort No comparison group Total n = 41	FQHC in the Bronx, N.Y., USA Urban	Team—based care between pharmacist and physician to jointly induce and monitor patients treated with BP Model(s): *Coordinated Care*, *Multi-disciplinary Care*, *Shared Care* **Duration**: 26 mo **Medication**: Buprenorphine	**PCP**: General Internist **Pharmacist** **Patient** **Social Worker**	**PCP**: collaborated with the pharmacist to induce patient on BP as well as prescribe and monitor patient progress **Pharmacist**: monitored and observed patient induction on BP; held joint phone/appointment visits with patient as needed **Social worker**: provided routine care as needed, though not required with program	*Staff*: PCP worked with pharmacist to induce patients onto BP Provided psychosocial, routine counseling as needed (i.e. motivational interviewing) BP dispensed on-site by the pharmacist *Patient*: All patients met with physician and/or pharmacist for visits and provided urine samples as requested	N/A	Retention: 70.7% at 90 days 90-day retention in treatment as confirmed by medical records Results: 29 (70.7%) were retained in treatment at 90 days	Not discussed	17 Fair
Cunningham et al (2011)	QE No comparison group Total n = 79	Community Health Center in the Bronx, N.Y., USA Urban FQHC	Multi-disciplinary Care Model with patient-centered home-based induction of BP vs. standard of care office-based induction Model(s): *Coordinated Care*, *Multi-disciplinary Care* **Duration**: 6 mo **Medication**: Buprenorphine	**PCP** **Pharmacist**	**PCP**: prescribed and monitored patient in either home induction or office-based induction; PCP also available to answer questions/concerns throughout induction and maintenance for patients **Pharmacist**: dispensed BP to patients from on-site pharmacy	*Staff*: PCP either induced patients in office-based setting or provided patients for patient-induced take home induction with kits and BP education prior to induction All prescriptions and dispensing provided by pharmacist at on-site pharmacy *Patient*: All patients met with physician and/or pharmacist for visits and provided urine samples as requested Home-induced patients were given kit and instructed to follow all directions	Home based induction kit: instruction sheet & BP Six sections explaining contents of the kit, when to start taking BP/NX, things not to do, how to take BP/NX, plans to guide treatment and facilitate follow-up, and a log to track medications taken	Retention: N/A Self-report of opioid use in previous 6 months Results: Among all participants, opioid use declined from 88.6% at baseline to 42.0% at 1 month, 33.3% at 3 months, and 27.3% at 6 months Opioid use and any drug use consistently declined at each period in patient-centered home-based inductions, not in standard-of-care office- based inductions	Not discussed	20 Good
DiPaula& Menachery (2014)	Observational Cohort No comparison group Total n = 12	Maryland, USA PCC in urban health department Urban	Coordinated care with collaboration between physician and psychiatric pharmacist Model(s): *Coordinated Care*, *Multi-disciplinary Care* **Duration**: 12 mo **Medication**: Buprenorphine	**PCP**: internist **Pharmacist**: specialized in psychiatry **Medical Assistant**	**PCP**: induced patients on BP, followed-up with pharmacist and patient to confirm and document treatment **Pharmacist**: Met with patient initially, followed-up weekly/monthly **Medical Assistant**: collected urine tox, took vitals	*Staff*: In initial visit, pharmacist met with patient to discuss: substance use, mental, and physical history as well as review clinical procedures and complete treatment contract with patient Physicians spent ~30 minutes after confirming treatment plan and discussed program with patient *Patient*: Attended all scheduled appointments, adhered to prescription and treatment contract	Patient contract	Retention: 73% at 12 mo. Substance abuse discovered via urine tox screens Results: 98% positive for BP and negative for other substances	Physicians favored the take-home BP induction method vs. traditional long-term maintenance	18 Fair
Doolittle & Becker (2011)	Observational Cohort No comparison group Total n = 228	New Haven, CT, USA Urban Internal medicine/ pediatrics PC practice	Physician-centric model where patients were self-referred, OUD care was provided within the practice with BP in conjunction with other comorbidities Model(s): Physician-Centric **Duration**: tailored to patient, 4 year study **Medication**: Buprenorphine	**PCP**: no extra training in addiction medicine	**PCP**: counseled patient about opiate withdrawal and the mechanism of action of BP in patient-centered language	*Staff*: "Buprenorphine contract": patient agreed to attend all appointments, submit regular urine drug tests, and not receive early refills of BP until next appointment 16 mg dosing with home induction and shared decision-making on length of treatment *Patient*: Self-referred to clinic, met with 1–2 PCP for complete history/physical Patient has 1 week follow-up monthly appointments, and PCP on call via phone if "dope sick” or for questions and concerns	Patient contract	Retention: N/A Withdrawal, urine test, cocaine test, and treatment of comorbidities 82% negative urine drug screen 92% negative for cocaine, 88% positive for BP	Home induction helped in ameliorating potential barriers (i.e. clinic resources and time) for providers	15 Fair
Drainoni et al (2014)	QE, Comparison group (Infectious Disease clinic vs. General Internal Medicine clinic) Total n = 265	Boston, MA Urban, primary care setting 2 clinics (1 infectious disease and 1 general internal medicine) were utilized in the FAST PATH program	FAST PATH team-based model of integrated care developed by a physician, nurse, and addiction counselor case manager team that used BP in PC with addiction treatment Model(s): *Coordinated Care*, *Multi-disciplinary Care* **Duration**: N/A **Medication**: Buprenorphine	**PCP** **RN** **Addiction counselor case manager**	**FAST PATH Team**: weekly meetings to discuss all services provided (individual tasks not discussed)	*Staff*: Provided ongoing primary care, medication assisted treatment when indicated (i.e. BP/NLX), HIV risk reduction counseling, individual and group counseling, referral to additional SUD treatment Aim was to expand/enhance treatment of alcohol and drug dependence among HIV infected and at-risk patients within PC settings 1 hour focus groups conducted by program manager and evaluator, semi-structured interview guide *Patient*: Attendance and participation as well as adherence to prescription	Patient contract	Retention: N/A Patients felt most strongly about their interactions with program staff Nonjudgmental, caring attitudes were highly valued Positively identified feature was group counseling format, but patients had mixed feedback on optimal content of counseling sessions Group care management to address holistic individual and individual needs	In FAST PATH the RN/counselor took ownership of the program—cited as key component to success of the program	15 Fair
Drucker et al (2007)	Observational Cohort No comparison group Total n = 14	Lancaster, PA, USA Rural PCC	“Lancaster Model”: PCP and community pharmacist worked collaboratively in sharing patient care Model(s): *Coordinated Care*, *Multi-disciplinary Care* **Duration**: 24 mo, 12 mo follow up **Medication**: Methadone	**PCP**: general internist with experience in addiction treatment **Community Pharmacist**	**PCP**: provided initial rx and act as case manager for patients; responsible for meeting with patient at least once a month **Pharmacist**: observed patient taking methadone at clinic, provided them with take-home rx, and communicated patient status and updates with PCP	*Staff*: PCP was responsible for meeting with patient and providing counseling as needed as patient’s case manager Pharmacist observed methadone induction and provided take-home doses and communicated with PCP after each observed dosage Pharmacist tracked rx’s and logged patients’ rx bottles *Patient*: Attendance and participation as well as adherence to rx; patient provided urine tox when requested and returned empty methadone bottles to pharmacist when refilling	Logs for rx’s and bottle-return monitoring	Retention: 86% at 12 mo. Retention in treatment, concurrent drug use, and patient and provider satisfaction Results: 10 patients remained at end of study period, illicit drug use was not a significant issue for population given urine tox results, overall patient satisfaction was good (complaints of distance from PCP, hours since most all patients worked, and other patients trying to sell them drugs in waiting room)	Provider satisfaction: overall very good, staff felt that the bottle returns were not necessary	17 Fair
Ezard et al (1999)	Observational Cohort No comparison group Total n = 195	Victoria, Australia Urban PC practices within the community	Community based service delivery in which patients were prescribed methadone via PCP then received daily dose from pharmacist at a separate site Model(s): *Coordinated Care*, *Multi-disciplinary Care* **Duration**: N/A **Medication**: Methadone	**PCP** **Pharmacist**	**PCP**: prescribed the methadone **Pharmacist**: supervised the daily dispensing of methadone	*Staff*: PCP prescribed the methadone which is then dispensed at a separate pharmacist daily *Patient*: Attendance and participation as well as adherence to prescription	N/A	Retention: 73% at 12 mo. Results: 85% reduction in substance use	Not discussed	16 Fair
Fiellin et al (2002)	QE Comparison group (Medical management vs Medical management + counseling) Total n = 14	New Haven, CT, USA Urban Academic PCC	Patients received MM (medical management) from nurse staff 3x a week with trained RN covering: review of recent drug use or abstinence efforts review of attendance at self-help groups support for drug reduction/abstinence brief advice on how to achieve or maintain abstinence 3x week urine sample collection Model(s): *Coordinated Care*, *Multi-disciplinary Care* **Duration**: 13 weeks **Medication**: Buprenorphine	**PCP**: general internists **RN**: Nursing staff with no prior experience in substance abuse **Psych**: PhD psychologist	**PCP**: met with patients to assess progress once a month and supervised nursing staff **Nurses**: conducted the weekly counseling, met with patients 3x weekly for brief counseling, met with PCP and psychologist to review	*Staff*: Nurses recruited from center's staff had no prior experience in substance abuse treatment Training in MM via designed manual and 3x 1 hour sessions on heroin dependence, BP, and counseling Weekly review of counseling issues with supervising PCP and doctorate level psychologist *Patient*: Patients received MM, manual guided treatment 3X weekly sessions with RN for ~20 min, and met monthly with PCP for ~20 minutes	Medical management guide	Retention: 79% at 13 wks. Illicit opioid use assessed by urine toxicology and treatment retention Positive urine toxicology results reduced significantly from 95% to 25% over 13 wks*	Physician and psychologist provided support for nursing staff, but no real discussion on provider outcomes or perception	23 Good
Fiellin et al (2004) *Assessed from Fiellin et al (2001)*	RCT (Fiellin et al, 2001) Comparison group (office-based treatment vs. opiod-treatment program) n = 6 physician interviews Total n = 46 (PCP = 22 vs. 24 in SC)	New Haven, CT, USA Urban 2 Academic PC practices 1 suburban based practice	Evaluated efficacy of OBOT- M vs. continuing treatment over 6 month follow-up largely coordinated by nurses Evaluated the OBOT-M efficacy via Randomized Control Trial from Fiellin (2001) Model(s): *Coordinated Care*, *Multi-disciplinary Care* **Duration**: 6 mo **Medication**: Methadone	**PCP**: all general internists, 4/6 certified in addictions medicine **RN**: nursing staff, and other office personnel	**PCP**: during initial visit: reviewed the patient’s medical and substance abuse history and treatment records from the OTP, performed a physical exam, and discussed components of OBOT-M with patient Mandatory 2x 4 hr training sessions **Nurses**: main responsibility was coordination of care in terms of scheduling physician visits with patients and collecting urine specimens for drug screens	*Staff*: Physician training of opioids, methadone maintenance, role of psychosocial treatment Patients were given oral dispense of methadone once weekly then given 6 day supply of liquid methadone, coordinated by RN Additional in-service training was provided at the office for nursing staff and other office personnel *Patient*: Patients scheduled to have 1x mo 30 min visits designed as counseling sessions to look for relapse, medication issues, health promotion, and participation in self-help or relapse prevention activities Patients were given oral dispense of methadone once weekly then given 6 day supply of liquid methadone Each patient had 1 medication dispensing visit per month at which they were asked to provide a urine for toxicology analysis (random urine screen all other visits)	Training & Resource Guide (developed specifically for program) Monthly on-site chart audits to assess MD adoption Med transfer logs to track receipt/ return of bottles	Retention: N/A Logistics of dispensing, the receipt of urine toxicology results, difficulties arranging psychiatric services, communications with the opioid treatment program, and non-adherence to medication as problematic *From Fiellin et al (2001)*: No statistically significant differences between primary care versus narcotic treatment program for illicit opiate use. PCP patients did think the quality of care was excellent compared to narcotic treatment programs.* 50% of OBOT-M patients vs. 38% of control had self-report or urine tox for positive illicit drug use Ongoing illicit substance use (defined as clinical instability) found in 18% of OBOT-M patients vs. 21% in control 73% of OBOT-M patients thought quality of care was “excellent” vs. 13% of control	Clinical management issues: charting certain findings (i.e. positive urine drug screens), incorrect methadone bottle logs, reformatting logs, difficulty referring patients to psychiatric services, problems with patient's medication adherence, and unnecessary required counseling for patients with prolonged abstinence Training adequately prepared MDs	19 Fair
Fiellin et al (2006)	RCT Comparison group (standard medical management + 1 or 3x medical dispensing vs. enhanced medical management + 3x medical dispensing) Total n = 166	New Haven, CT, USA Urban Academic PCC	Patient centered model with standard or enhanced medical management given to individual patients. 3 treatment arms: standard MM + 1x wk medication dispensing standard MM + 3x wk medication dispensing, enhanced MM + 3x wk medication dispensing Overall goal to assess differences in counseling and medication dispensing for BP patients Model(s): *Coordinated Care*, *Multi-disciplinary Care* **Duration**: 24 weeks **Medication**: Buprenorphine	**PCP** **RN**: Nurses with no previous experience treating addiction **Psych**: psychologist	**PCP**: met with patients monthly for 20 minutes **Nurses**: dispensed the BP & facilitated weekly manually guided standard or enhanced MM to individual patients **Psychologists**: met weekly with physician and nurse to review counseling	*Staff*: Standard MM: brief, manually guided, medically focused counseling, ~20 min long Enhanced MM: similar to standard but longer session, 45 min long MM topics included: recent drug use and efforts to achieve or maintain abstinence, urine analysis results, advice for abstinence achievement/ maintenance Nurses dispensed the BP, and were the facilitators for the counseling sessions The nurses, physician, and psychologist met monthly to discuss the counseling sessions *Patient*: Patients met 3x week for 2-week induction/stabilization period and progressed to 16 mg (max 20–24 mg) BP daily for 24 weeks Take-home medication provided to patients for days on which they did not receive BP from office Adhere to treatment assignment, provide urine samples, and attendance to all follow-up	Recorded audio for counseling Electronic caps of medication bottles (Medication Event Monitoring System) Caps contain micro-processors that record, but don’t display date and time each bottle is opened	Retention: N/A No statistical significance in negative urine screens, maximum consecutive weeks of abstinence, reduction in frequency in illicit drug use or proportion of patients remaining in study between groups Overall significant reduction in illicit opioid and cocaine use Treatment satisfaction was significant with treatment group: higher satisfaction with standard MM and 1x wk medication dispensing*	Not discussed	21 Good
Fiellin et al (2013)	RCT Comparison group (physician management vs. physician management + cognitive behavioral therapy) Total n = 141	New Haven, CT, USA Urban PCC	Patient centered model with randomization to 2 groups and followed over 12 weeks 2 treatment arms: 1) Physician Management 2) CBT Model(s): *Coordinated Care*, *Multi-disciplinary Care* **Duration**: 24 weeks **Medication**: Buprenorphine	**PCP** **RN**: nursing staff **Psych**: Masters & PhD Clinicians for counseling	**PCP**: provided the physician management **Psych**: Masters & PhD clinicians provided CBT study arm	*Staff*: Physician Management (PM): manual guided, medically focused, 15–20 minute weekly counseling session for first 2 wks, every other week for 4 wks, and then monthly Topics discussed: recent drug use and efforts to achieve or maintain abstinence, urine analysis results, abstinence advice on achievement /maintenance advice, review of medical/psychiatric symptoms, assess social, work, and legal function, group attendance, and urine screen results CBT: manual guided, weekly 50 min sessions provided for first 12 wks of treatment by trained masters and PhD clinicians Main components: performing functional behavior analysis, promoting behavioral activation, identifying/ coping with drug cravings, enhancing drug-refusal skills, enhancing decision-making about high-risk situation, and improving problem-solving skill *Patient*: Patients met with either the PCP or underwent CBT for counseling depending on treatment group Adhere to study protocol and attend counseling; meet with nursing staff 3X wk for first 2 wks	CBT manual adapted (from cocaine use) for study Recorded audio & videotape of CBT sessions	Retention: 45% in PM; 39% in CBT at 6 mo. Self-reported frequency of illicit opioid use, maximum number of weeks abstinent from illicit opioids evidenced by urine tox and self-report Significant reductions from baseline in both treatments from 5.3 average days of opioid use to 0.4* No significant differences between groups Time had significant impact on retention rates*	However, PCPs cite lack of available ancillary psychosocial services a barrier For some patients psych may not be necessary	22 Good
Gossop et al (1998)	QE Comparison group (primary care vs specialty care clinic) Total n = 452 SC n = 297; PC n = 155	UK: National Health System Urban Community-based specialist clinic or GP setting	GPs or Specialists provided methadone maintenance to patients Model(s): *Coordinated Care*, *Multi-disciplinary Care* **Duration**: 6 mo **Medication**: Methadone	**PCP**: General practitioners **SCP** **Pharmacist**	**PCP**: responsible for prescribing medications **Pharmacists**: Responsible for supervision of medication in some clinic settings No discussion of who was performing the interviews	*Staff*: Processes differed between groups At the program level, differences were found in the manner in which methadone was dispensed Fewer GP agencies (57%) than clinics (75%) prescribed daily dispensing of methadone 6 of the 8 clinics used supervised dispensing procedures (on site or supervised by a retail pharmacist) Supervision (to be provided at retail pharmacies) was used less often by GP agencies (14%) *Patient*: Adhered to prescription as well as follow-up appointments	N/A	Retention: 66% of GP patients; 60% of SC at 6 mo. Over 50% reduction in heroin use for both groups No statistical difference between groups	Concerns highlighted include: the perception that the methadone maintenance patients may be difficult and upset other patients within the clinic	19 Fair
Gossop et al (2003)	QE Comparison group (primary care vs specialty care clinic) Total n = 240 SC n = 161; PC n = 79	UK: National Health System Urban SC clinic or PC setting	OBOT-M with 5 of the 7 PC sites using coordinated care models (physician prescribing and clinic providing counseling services) Model(s): *Coordinated Care*, *Multi-disciplinary Care* **Duration**: 12 mo, follow up 24 mo **Medication**: Methadone	**PCP**: General practitioners **SCP** **Pharmacist**	**PCP**: responsible for prescribing medication and provided medical care as required **SCP**: provided counseling services **Pharmacists**: direct supervision within retail pharmacy Data on patient outcomes was collected using interviews, but not discussed who collected them	*Staff*: Supervision used in prescribing Type of methadone (tablet vs. liquid) varied between SC vs. PC setting *Patient*: Adhered to prescription as well as follow-up appointments with PCP	N/A	Retention: 61% of PC; 53% of SC at 12 mo. Illicit drug use, drug injecting behaviors, alcohol use, crime, physician and mental health problems Significant reductions heroin use, non-prescribed methadone and benzodiazepine uses and stimulants Significant differences between PC and SC for: non-prescribed benzodiazepines and stimulants usage, frequency of alcohol use and psychological health Significant differences in psychological health, stimulant use, and non-prescribed benzodiazepine use between groups	Not discussed	17 Fair
Gruer et al (1997)	Observational Cohort No comparison group Total n = 1971	Galsgow, Scotland, U.K. Urban PCC	Glasgow Scheme is a service led by former PCP with experience in OUD patient population Staffed by 4 teams of 2–3 specialist RNs working with MDs that each cover 25% of area covered by health board Model(s): *Coordinated Care*, *Multi-disciplinary Care* **Duration**: 12 mo **Medication**: Methadone	**PCP**: General practitioner **RN**: trained nurse with drug counseling **Community pharmacist** **Drug counselor**	**PCP**: provided prescription and attended drug misuse training at least twice a year, completed the opiate treatment index for each patient, routine care as needed **Nurses**: trained in counseling and provide services as necessary **Pharmacists**: supervised dosage **Drug counselors**: provided patient counseling	*Staff*: Trained team of PCP, Pharmacist, Drug counselor and RN in methadone and drug use, misuse, and abuse PCP given 5–20 patients Specific scheme guidelines for assessing and treating patients Only allowed to prescribe oral methadone 1 mg/ml Daily methadone self-administration with supervision under community pharmacist Patients with coexisting benzodiazepine dependence prescribed reducing doses of diazepam or nitrazepam Temazepam forbidden Brief details of each patient's attendance noted and health and social circumstances recorded initially and every 6 mo2 *Patient*: All patients received regular additional counseling from a drug counselor or trained RN After assessment of the patient the service will usually initiate treatment only if the general practitioner agreed to participate in ongoing care thereafter A written contract is created with the patient Stabilized patient ongoing care returned to PCP with service still available for advice	Patient Contract	Retention: 60% at 12 mo.	Beneficial in establishing the Glasgow Drug Problem Service Scheme provides detailed guidance on methadone maintenance therapy Improves managing patients Positive continuing education for PCPs	13 Poor
Gunderson et al (2010)	RCT Comparison group (observed vs. unobserved office induction) Total n = 20	NYC, NY, USA Urban PC clinic	Patient centered model with unobserved vs. observed induction of BP Model(s): *Coordinated Care*, *Multi-disciplinary Care* **Duration**: 12 week follow up **Medication**: Buprenorphine	**PCP**: Internist with BP experience **Pharmacist** **Study Personnel**	**PCP**: BP induction, provided routine care, and provided phone support to induced patients **Pharmacists**: dispensed BP **Study personnel**: picked up BP from pharmacy and stored in locked medicine cabinet and phone calls to patients	*Staff*: Target daily maintenance dose is 12–16 mg with max of 32 mg Weekly clinical visits during 4-week induction and stabilization phase then decreased to monthly visits Urine toxicology with BP-specific immunoassay performed at all clinical visits as well as Research visits occurred every 4 wks (urine screen, self-reported substance use assessed, research scale administered) *Patient*: Patients receiving clinical dosage suggested to use psychosocial services and counseling support but not enforced Unobserved induction of BP/NX vs. office based induction	Subjective Opioid Withdrawal Scale (SOWS) administered via phone	Retention: 45% at 3 mo. Successful induction one week after initial clinic visit Similar induction rates between groups 60% successfully inducted in both groups 30% experienced prolonged withdrawal 40% stabilized by week 4 No statistical significance in phone calls for home-induced patients in office vs. unobserved induction	Not discussed	21 Good
Haddad et al (2014)	Observational cohort study No comparison group Total n = 266	Meriden and New Britain, CT, USA Urban FQHC's	Comprehensive, coordinated care between NP, PCP, and Psych to deliver PC opioid maintenance therapy through BP while treating comorbidities Shared care between NP, PCP, and Psych to oversee day-to-day clinical work Model(s): *Coordinated Care*, *Multi-disciplinary Care*, *Shared Care* **Duration**: N/A **Medication**: Buprenorphine	**PCP** **RN**: NP and nursing staff **Pharmacist** **Psych**: psychiatrist **Behavioral counselor** **medical assistant**	**Physicians and psychiatrists**: prescribed BP PCP could use the Electronic Health Record's pop up feature **NP and nursing medical assistants**: routine clinical care within their scopes of practice **Behavioral health workers** (psychiatrist and behavioral health counselors): used a health template during encounters	*Staff*: PCP and Psych can prescribe BP, but NPs cannot PCPs have a NP, an assigned nurse, and a medical assistant Psych has the behavioral counselors for assistance *Patient*: All patients contacted with appointment reminders and with test results by phone and/or mail	Phone calls and/or email to patients Use of electronic health records	Retention: 71.8% at 3 mo. Examined 9 QHI (HIV, HBV, HCV, Syphilis, hypertension, hyperlipidemia, cervical, breast, and colorectal cancers) Achieving at least an 80% QHI score was positively and independently associated with at least 3-month BMT retention & BMT prescription by PCP rather than addiction psychiatric specialists	Most PCPs did not use EHR pop up feature	18 Fair
Hersh et al (2011)	Observational Cohort study No comparison group Total n = 57	San Francisco, CA Urban community based, PC office clinics	Patient care delivered in 3 model sites *OBOT Buprenorphine Induction Clinic (OBIC)* initiated all BP treatment via part-time PCP and NP. Phone screening before scheduled evaluation/induction visit (Monday or Tuesday AM) *Community Behavioral Health Service (CBHS)* provided ongoing dosing with optional observed dosing *Community Treatment sites*: 2 PC clinics and 1 outpatient private dual-diagnosis group practice (provides outpatient mental health and addiction counseling services) Model(s): *Coordinated Care*, *Multi-disciplinary Care* **Duration**: 12 mo, follow up 1 mo, 3 mo, 6 mo **Medication**: Buprenorphine & Methadone	**PCP**: some physicians trained in BP while others not **RN**: NP **Pharmacist** **Psych**: psychiatrists	**Physician** and **NP** at the OBIC: induced the patient on BP and stabilized to maintenance dose **Pharmacists** at CBHS: supervised dosing for all patients	*Staff*: Centralized induction clinic for BP staffed by PC and NP Patients initially given full physical/mental health assessment, BP and pilot program education, informed consent, and release of information Induction dose of 2-4mg on first day with option 2-4mg additional to reduce withdrawal and titrated to 12-16mg/daily of BP Visits 4–5 days first week with reduction to weekly visits *Patient*: Observed dosing for take-homes (day 3 of treatment) Informal methadone maintenance program helped refer patients to BP pilot Minimum requirements included: monthly counseling, quarterly urine toxicology screening, and quarterly visits with the treating PCP	Phone Screening No written prescriptions given to patients OBOT electronic database	Retention: 61% at 12 mo. Over 50% reduction in positive urine screens for methadone and morphine in first 30 days of treatment* Significant increase in paid-working days and decrease in reported drug problems No significant changes in medical or mental health problems Positive patient perceptions of the program (75% felt comfortable with PCP, 15% thought OBIC was very difficult, majority felt PCP knowledgeable and highly valued monthly counseling	Over time community PCPs grew increasingly comfortable leading to fewer pharmacy visits average of 2–3 visits per week to weekly, every other week, or monthly visits) OBOT database helped to facilitate communication between the PCPs, pharmacists, and counselors	20 Good
Kahan et al (2009)	Observational cohort study No comparison group Total n = 200	Toronto, Ontario, Canada Urban Academic family medicine unit	Multi-disciplinary Care program with nurse clinician, family therapist, 6 PCPs, clinical fellow in which patients receive brief counseling intervention, outpatient medical detox, pharmaco-therapy & follow-up Model(s): *Coordinated Care*, *Multi-disciplinary Care*, *Shared Care* **Duration**: 4 mo **Medication**: Methadone	**PCP** **RN**: nurse clinician **Psych**: family/ addiction therapist	**PCP**: initial physical assessment, pharmacotherapy selection and induction **NP**: not stated **Addiction therapist**: initial assessment of patient including demographic, drug/alcohol use	*Staff*: Patient first assessed by addiction therapist and then by PCP Consultation note faxed to PCP with brief history, diagnosis, and treatment recommendations Pharmacotherapy determined by PCP and consultation note *Patient*: Patients received brief counseling session and outpatient medical detoxification program After completion, patient reassessed	Telephone follow-up with patients with the research assistant Faxing of consultation note to PCP	Retention: N/A Changes in self-reported substance use from interviews at intake and 3–4 months after initial office visit 13/29 OUD patients had statistically significant decreased MME and decline in mean number of drinks* 31% of participants participated in Alcoholics Anonymous or formal addictions treatment	Not discussed	20 Good
Lintzeris et al (2004)	RCT Comparison group (methadone maintenance vs, buprenorphine maintenance + option to transfer to methadone) Total n = 158	SC (1) and PC clinics (18) and Community Pharmacies (30) Melbourne and Victoria, Australia Urban	Compared delivery methods by specialist vs. community-based service providers via BP or Methadone Model(s): *Coordinated Care*, *Multi-disciplinary Care* **Duration**: 12 mo, follow up 3, 6 mo **Medication**: Buprenorphine & Methadone	**PCP**: General practitioners without BP training **Pharmacist**	**PCP**: prescribed BP **Pharmacists**: supervised the dosing	*Staff*: PCP prescribed BP and coordinated patient care Supervised dosing by pharmacist *Patient*: Patients assigned to either control group (conventional methadone maintenance treatment program) or experimental group (BP treatment with option for methadone transfer) Subjects followed over 12 mo period with treatment coordinated by prescribing PCP Required monthly meetings and optional counseling services available Daily supervised induction of sublingual BP tablets (2 and 8 mg) with flexible doses Once stabilized, transition to alternate-day or 3-day dosing	N/A	Retention: 70% in PC; 77% in SC at 3 mo.	Create readily available set of BP guidelines suited for community settings Medium-dose transfers from methadone to BP were difficult to conduct in community settings Pharmacies addressed problems of diversion and delays in dosing by crushing BP tablets and administering sublingual BP powder	20 Good
Lucas et al (2010)	RCT Comparison group (clinic based buprenorphine vs. referred treatment) Total n = 93	Baltimore, MD, USA Urban The Johns Hopkins HIV Clinic (single center) where BP treatment was integrated into an HIV primary care clinic	Multi-disciplinary care between 2–5 BP PCPs, social worker, substance abuse counselor, and nursing staff Model(s): *Chronic Care*, *Coordinated Care*, *Multi-disciplinary Care* **Duration**: 12 mo **Medication**: Buprenorphine	**PCP** **RN**: LPN trained in substance abuse **Substance abuse counselor**	**PCP**: collaborated with LPN and substance abuse counselor, oversaw prescribing, and met with patients for follow-up **LPN**: managed the patients **Substance abuse counselor**: met with patients to schedule follow-up and induction education	*Staff*: Social worker and registered nurse ran the case management program, coordinated appointments, and assisted with overcoming barriers to adherence PCP met with patient after 4 wks *Patient*: Patient initial 2-day BP induction (3x BP daily dose) & progressed to clinic treatment until stabilized Unstructured counseling provided, urine drug tests, and take-home supplies of BP provided each visit	N/A	Retention: N/A Initiation and long-term receipt of opioid agonist therapy, PCP visit attendance, RNA CD4 cell count changes, and use of antiretroviral therapy Patient satisfaction higher in OBOT setting Clinic-based patients lower levels of reported injection use and Hep. C co-infection 78% of referred patients met with case manager (average 3 meetings) 64% started methadone or BP	Not discussed	23 Good
Michelazzi et al (2008)	Observational Cohort No comparison group Total n = 33	Trieste, Italy Urban GP’s outpatient office	OBOT-M Model(s): Physician-Centric **Duration**: 429 days (+/- 273 days) **Medication**: Methadone	**GP**	**GP**: prescribed methadone, met with patients at least once weekly and provided routine care	*Staff*: GP required to meet with patient routinely to discuss progress, provide support, and monitor patient within the outpatient setting *Patient*: Patient was responsible for completing each of the evaluations which included: substance abuse, psychosocial health, personal data, urine analysis, and other pertinent medical histories	N/A	Retention: 78% at 12 mo. Statistically significant decreases in drugs of abuse from baseline to endpoint	Not discussed	17 Fair
Moore et al (2012)	RCT Comparison group (physician management only vs. physician management + cognitive behavioral therapy) Total n = 58	Urban Adult PCC affiliated with the hospital system New Haven, CT, USA	Office based BP treatment with added CBT Model(s): *Coordinated Care*, *Multi-disciplinary Care* **Duration**: 14 weeks **Medication**: Buprenorphine	**PCP**: trained in internal medicine **RN**: Registered Nurse **Psych**: Therapist	**PCP**: BP treatment, reviewed weekly therapists taped session to ensure competence and adherence to proper quality of care, meet with patients **Therapists**: led the CBT sessions	*Staff*: PCP led BP treatment and therapists led the CBT sessions PCP reviewed weekly taped therapy session to ensure competence and adherence to quality of care *Patient*: Final 2 wks PCP and patient established agreement for final tapered dosing Patients attended scheduled visits before induction, after induction, and at the end of month (total 5 visits)	N/A	Retention: PM+CBT: 19%; PM: 26% at 14 wks. Increase in negative urine screens* Decrease in opioid use* Physician management only had highest %negative urine screens & lowest % opiod use* CBT attendance associated with increased negative urine screens & abstinence length * Overall patient satisfaction was high	Difficulties arose with CBT Difficult finding office space, transportation and parking Problems coordinating care team and increased treatment costs Adaptive/ stepped-care model of treatment hypothesized to help high risk patients	21 Good
Mullen et al (2012)	Observational Cohort No comparison group Total n = 1269	Ireland Urban PC &. SC centers	Coordinated care between multidisciplinary team in SC and community centers with SC Model(s): *Coordinated Care*, *Multi-disciplinary Care* **Duration**: 12 mo **Medication**: Methadone	**PCP** **RN**: Registered Nurse **Psych**: Psychiatrists **Counselors**	**PCP**: provided methadone maintenance to patients **Psych**: provided psychosocial support as needed and lead SC centers	*Staff*: PCPs trained by the Irish College of General Practitioners PCP can choose to prescribe and deliver methadone PCPs are part of care team for addiction treatment in Ireland Methadone maintenance by PCP is central to drug treatment system No standard clinical practice guidelines in Ireland so UK guidelines followed *Patient*: Attend all scheduled follow-up visits, adhere to methadone dosage Drug users transferred from community drug treatment centers to PC once stabilized	N/A	In SC and PC combined, retention: 61% at 12 mo. In SC and PC combined, treatment retention at 12 mo associated with age, gender, facility type, and dose Age and gender no longer significant when adjusted for other variables Patients attending SC site were 2x likely to leave program with 12 mo vs PCP site Biggest predictor of treatment retention was methadone dose regardless of type of treatment facility Patients receiving <60 mg of methadone were 3x more likely to leave treatment	SC has more severe patient population	22 Good
O'Connor et al (1998)	RCT Comparison group (primary care vs. specialized care) Total n = 46	New Haven, CT Urban PC setting in Central Medical Unit affiliated with Yale Substance Abuse Treatment Unit vs. specialty care clinic in Legion Avenue Methadone Maintenance Program	Manual-guided clinical management with team-based approach in SC clinic with PCP Model(s): *Coordinated Care*, *Multi-disciplinary Care* **Duration**: 3 mo optional 10 week extension **Medication**: Buprenorphine	**PCP**: General Internists **RN**: NPs **Physician Associates** **Counselors**	**PCP**: prescribed treatment, performed initial assessment, followed patient throughout study (if PCP vs. SC) **NP**: ran semi-structured weekly group therapy **Counselors**: in SC provided substance abuse counseling and services	**PC clinic**: *Staff*: Same PCP followed patient throughout the entire treatment NP ran 50 min semi-structured weekly group therapy sessions *Patient*: Patient required to attend initial referral and full clinical assessment, shared decision-making when establishing goals, medical and substance abuse history reviewed, PCP educated about risks/benefits of treatment, and mandatory weekly group therapy Followed-up weekly for 20 min (urine screen, treatment review, adapt goals to current status) **SC clinic**: *Staff*: SCP prescribed treatment and initial dose assessment and patient had substance abuse counselor *Patient*: Patient attended clinic 3x week for prescription, urine screens, and self-reported follow up reports Patient attended mandatory weekly group therapy and optional 1x mo individual counseling session	N/A	Retention in treatment and urine toxicology Retention: 78% in PC; 52% in SC at 3 mo. PC patients (63%) had lower rates of opioid use than SC (85%)* PC higher 3+ week abstinence (43%) vs. SC (13%)* Higher patient satisfaction in PC	Properly trained General internists can provide OUD treatment Number of visits impractical for a PC workload Decreased prescription frequency can diminish long-run retention in treatment PCP remained willingly kept OUD patients in PC setting Reimbursement method for these services in PC is lacking (capitated Full-risk managed care plan possible solution)	21 Good
Ortner et al (2004)	Observational Cohort No comparison group Total n = 60	Austria Urban SC initiation with transfer of care to PC centers	Coordinated Care between SC and PC with long term PC care Model(s): *Coordinated Care*, *Multi-disciplinary Care* **Duration**: 3 mo **Medication**: Buprenorphine	**PCP**: in primary care setting with special training for opioid addiction **SCP**: in specialty care setting in short based SC induction and long-term treatment continuation in PC	**PCP**: prescribed BP and oversaw duration of care after induction **Pharmacists**: supervised daily intake of BP for first month	*Staff*: Induction was initiated by SCP and then care transferred to PCP Pharmacies helped oversee and monitor BP intake first month of PC treatment *Patient*: Patient transitioned to take home doses until stabilized Upon stabilization patient referred to PCP to continue maintenance treatment	N/A	Retention: 57% at 15 wks. (after completion of SC and PC segments) Urine samples for opioids, cocaine, and benzodiazepines, were positive in 28.9%, 19.6%, and 13.1%, respectively Self-report for depression and withdrawal symptoms: depressive symptoms never reached clinical relevance Withdrawal symptoms decreased within first week (patient in SC)* No significant differences between SC and PC retention rates between 3 week SC period and 12 week PC period or mean bupenorphine dose Across both SC and transition to PC, significant reduction in opioid use and cravings for heroin and cocaine*	PCP active involvement in treatment needed for patients to receive adequate care Special training programs about OUD needed for PCPs	19 Fair
Roll et al (2015)	Cross-sectional observational study No comparison group Total n = 28	Revere, M, USA Urban Safety net primary care center at Revere Family Health Center	Shared medical appointments model run by PCP and certified addictions nurse with patients treated with OBOT-BP Model(s): *Coordinated Care*, *Multi-disciplinary Care* **Duration**: 1 mo **Medication**: Buprenorphine	**PCP** **RN**: addictions certified registered nurse	**PCP**: provided general care **RN**: provided check-ins for patients and utilized structured educational models	*Staff*: PCP and nurse collaborated in providing care through shared medical appointments *Patient*: Patient attended 75 min sessions about 1-4x a month Patients self-reported life circumstances, current health status, and mental health status during each visit	N/A	Retention: N/A Patient satisfaction, management of other comorbidities, vaccination, housing improvement, time spent working, and resolution of legal cases Patients reported liking group visit format Patients in program gained increased coping skills, had more stable housing and less legal difficulties Shared medical appointments for OUD was highly acceptable	Possible improvements were increasing availability of groups outside of working hours and expanding range of patient educational modules	13 Poor
Ross et al (2009)	Observational Cohort No comparison group Total n = 190	Edmonton, Alberta, Canada Urban community-based PCC	A patient centered approach used to facilitate treatment through MM Model(s): *Coordinated Care*, *Multi-disciplinary Care* **Duration**: 1.5 mo **Medication**: Methadone	**PCP** **RN**: NP **Psych**: Mental Health Workers **Addiction Counselor** **Social Workers**	**PCP**: BP prescription as well as scope of care beyond NP and bridging patient **NP**: enrollment of physicals and routine care **Social Workers and Mental health workers**: mental health assessments, provided counseling, and linked to outside services	*Staff*: PCP had primary role in prescribing medication and coordinating follow-up care Staff provided additional services to patient throughout process PCP oversaw medical issues and prescribing BP beyond NP scope NP provided limited prescribing and enrollment physicals Social workers and mental health workers provided mental health assessment, individual patient counseling, and financial aid, housing, and social assistance *Patient*: Completed all required assessments and attendance to appointments	Clinical team accessible via phone 24/7 Faxing prescriptions Pharmacist contacted via phone to prevent prescription misuse Patient contract	Retention: N/A Types of medication used for bridging in patients waiting for methadone treatment 79% patients undergoing bridging used long-acting formulation 70% used MS Contin or Codiene Contin Bridging is good option for individuals forced to wait for treatment Meetings with PCP increased change of enhanced long-term care continuity of treatment	Barriers to care: cost of the clinic, prescription challenges Staffing expenses high Significant effort required to reduce misuse of prescribed medications	16 Fair
Sohler et al (2009)	QE Comparison group (office-based vs. home-based inductions) Total n = 115	Bronx, NYC, USA Urban community-based health center that provides PC	Chronic Care Model: focus on patients and relationship with physician The model was modified to address home induction Model(s): *Chronic Care*, *Physician-Centric* **Duration**: 30 days, follow-up 2 years post study initiation **Medication**: Buprenorphine	**PCP** **RN**	**PCP**: oversaw care for patients including BP prescribing, induction dosage, and patient follow ups **RN**: provided assistance as needed	*Staff*: PCPs helped determine patient eligibility for office-based versus home-based induction PCP available for contact outside of clinic hours via phone, but patients called infrequently *Patient*: Patients attended initial visit to determine BP treatment process (office-based vs. home-based induction) Patients with home-based induction had initial PC center visit and required follow up within 1 wk Shared decision making in long term maintenance plan Patient self-management highly encouraged	PCP available via phone For home-based inductions, patients given home-induction kit with instructions (explained contents, what to do, when to start taking BP, things not to do, how to take it, plans to guide treatment and facilitate follow-up, and a log to track meds taken),	Retention: 78.1% in OBOT; 78.4% in home-based at 30 days PC higher patient satisfaction than SC	Not discussed	21 Good
Tuchman et al (2006)	RCT Comparison group (office-based/community pharmacy dispensing vs. methadone maintenance treatment program) Total n = 26 Office-based practice/Community-pharmacy dispensing n = 14; control n = 12	Albuquerque & Santa Fe, New Mexico, USA Primary care settings (including women’s specialty health clinic) as well as community pharmacy	Office-based prescribing with community pharmacy integration for methadone maintenance patients with support from social workers Model(s): *Coordinated Care*, *Multi-disciplinary Care* **Duration**: 12 mo **Medication**: Methadone	**PCP**: 8 hours of didactic methadone maintenance training **NP**: 8 hours of didactic methadone maintenance training **Community Pharmacist**: 8 hours of didactic methadone maintenance training **Social Workers**: Masters level clinician	**Providers (4 PCP and 1 NP)**: provided continuous care for assigned methadone maintenance patients Responsible for faxing prescription to patient’s most convenient pharmacy **Pharmacist**: dispensed the methadone and oversaw the day’s methadone dosage **Social worker**: coordinated care and provided all psychosocial treatment	*Staff*: The provider team provided all clinical care and prescribed methadone and were responsible for ensuring patient’s prescription was faxed to most convenient pharmacy Pharmacist was responsible for dispensing methadone as well as observation of daily dose and dispensing of take-home dose according to PCP’s orders Social worker met with each patient for psychosocial treatment once a month *Patient*: Patients had regular urine toxicology tests, monthly counseling with the social worker and were required to adhere to prescription and all scheduled appointments	N/A	Retention: 100% in office based experimental group; 89% in MMT at 12 mo. Results: patients in the experimental group did as well or better than the control (routine methadone maintenance treatment program) Proportion of women continuing opioid use during study for experimental group was "significantly lower" in experiment group than control Pharmacy dispensing seen as positive given commentary, no statistics reported	Pharmacy dispensing was a critical factor in program: provided a positive environment for patient without any stigma and viewed as strength for rural settings	20 Good
Walley et al (2015)	Observational cohort study No comparison Group Total n = 265	Urban Academic HIV PCC and General Internal Medicine run clinic Boston, MA	Team of PCPs, NCM and licensed addiction counselor that collaborated to provide addiction care and patients had established treatment agreements with care teams Model(s): *Coordinated Care*, *Multi-disciplinary Care* **Duration**: 6 mo **Medication**: Buprenorphine	**PCP**: general internists **NCM** **Licensed Addiction Counselor**	**PCP**: provided addiction treatment and primary care as needed **NCM**: initiated face-to-face BP inductions and RN pill counts **Counselors**: provided motivational interviews via a 12-step program	*Staff*: The team provided an initial multidisciplinary addiction assessment by PCP, NCM, Counselor Addiction pharmacotherapy included BP/NX, acamprosate, disulfram with established treatment agreements Case management referred patients to methadone maintenance treatment and detoxification programs Weekly team meetings held before PCP clinical session to discuss patient coordination of care and treatment plan *Patient*: Patients had regular urine toxicology tests, weekly HIV counseling meetings by NCM, and individual and group counseling with addiction counselor	EMR Patient contract	Retention: N/A 60% had BP treatment 64% engagement by 6 mo, 49% had substance dependence BP treatment associated with engagement Self-reported depression baseline associated with substance dependence at 6 mo Housing status and polysubstance use not associated with engagement or substance dependence	Important for PCP to understand which patients more likely to engage Identify patients likely to have persistent substance use disorders Such knowledge helps target, tailor, and improve integration of addiction treatment and medical care	20 Good
Weiss et al (2011) *[22]* *Fiellin et al (2011)[23]* *Korthuis et al (2011)* *Korthuis*, *Tozzi*, *et al (2011)* *Egan et al (2011)* *Altice et al (2011)* [23–26]	Observational cohort study No Comparison Group Total n = 427 Included in analyses n = 303 Funded program n = 10	A group of Hospital Based HIV clinics across the United States Urban	Multi-disciplinary care model with comprehensive medical and social services available to all participants within the BHIVES program in which a "specialist" model of BP/NX treatment (limited number of PCPs oversaw entire pharmacotherapy process) was employed Evaluated the implementation of BHIVES model from Fiellin et al (2011): analyzed patient outcomes (retention in treatment, treatment process in terms of BP dosing, and illicit substance use, across the 9 sites Model(s): *Coordinated Care*, *Multi-disciplinary Care* **Duration**: 5 years (variable) **Medication**: Buprenorphine	**PCP** **NP** **RN**: registered nurse **Pharmacist** **Psych**: Psychologist **Social Worker** **Health Educator** **Substance Abuse Specialist**	**PCP**: oversaw entire pharmacotherapy process	*Staff*: Staffing varied among all participating sites Predominantly PCP led treatment process with support *Patient*: Care provided was non-punitive and offered opportunities that initiated conversations instead of dictating particular patient expectations Patients had BP treatment and normal induction	EMR Patient contract	*Weiss et al (2011)*: Evaluation and Support of programs to improve better understanding of BP/NX integration practices, services offered, staffing needs, PCP experiences/perceptions of BP/NX, perceived barriers and facilitators, sustainability measures, and recommendations for replication of integrated care program components Successful introduction of BMT program Many patients presented with multi-substance abuse and complex mental health comorbidities PCPs not in grant-funded programs adopted BP/NX tx more slowly BHIVES outcomes from *Fiellin et al (2011)*: Retention: 74% at 3 mo. BP patients 33% less likely to use illicit substances Treatment retention associated with female gender, black race, and greater number of years since HIV diagnosis *Korthuis et al (2011)*: 78.4% of patients receiving bup/nx remained on treatment at 3 mo, 72.7% at 6 mo, 62.9% at 9 mo, & 53.1% at 12 mo Mean summary quality score increased over 12 mo from 45.6% to 51.6% for bup/nx patients* *Korthuis*, *Tozzi*, *et al (2011)*: At 12 mo, average composite mental health-related quality of life (HRQOL) improved (38.3 to 43.4) and composite physical HRQOL did not change Bup/nx associated with improvements in HRQOL *Egan et al (2011)*: Patients satisfied with Buprenorphine/Naloxone and reported overall increased quality of life Counseling seen as an important component All patients strongly positive about integrated care model *Altice et al (2011)*: Retention on BUP/NX for 3+ quarters, significantly associated with increased ART initiating* Prescription of BUP/NX for 3+ quarters for patients on ART (at baseline) was not associated with statistically significant improvements in viral suppression and CD4 counts	Satisfaction with HIV & BP/NX integrated tx Challenges: Multi-OUD, mental health issues, poorly incorporating new procedures into practice, low psychiatric involvement Addiction med & OUD knowledge beneficial Complicated patients need outreach staff, case mgmt, & counseling Communication skills a positive	17 Fair

^a^Patient outcomes ranged from retention rate, increase in comorbidity screening, etc.

*statistically significant (p < 0.05) outcomes

### Definitions of SEIPS domains

The SEIPS 2.0 framework, previously used to evaluate system-level approaches to work systems, was used to evaluate the implementation of included study interventions. [14] The SEIPS model is a widely used healthcare human factors framework adopted by patient safety leaders and applied to multiple health settings including primary care clinics. The three human factor principles this model embraces are 1) evaluating performance from a systems orientation, 2) supporting person centeredness through designing work systems that best fit peoples’ capabilities, limitations and performance needs, and 3) focusing on design-driven improvements to develop structures and processes that enhance patient, provider and organizational outcomes. [14] This framework includes domains regarding the person, organization, technologies and tools, tasks, environment, process, patient outcomes, and employee and organizational outcomes of interventions [14] (Table 2). This framework allowed us to categorize the components across the included studies in a systematic way for better comparisons among interventions considering the heterogeneity of study outcomes and processes. Furthermore, SEIPS guided our assessment of the various components that were included in each model to identify what specific processes were impacted and who was involved.

**Table 2:** SEIPS model, current state, and areas for improvement.

SEIPS DOMAINS	DEFINITIONS	CURRENT STATE	AREAS FOR IMPROVEMENT
*ENVIRONMENT*	**Environment**: the physical environment and location of the system	8 countries globally (U.S., UK, Australia, Canada, Austria, France, Ireland, Italy) Highly variable setting Primarily health centers affiliated with academic institutions	Expand primary care interventions to more community health settings
*ORGANIZATION*	**Organization**: includes concepts such as relationships between healthcare workers and patients as well as coordination, collaboration, and communication between those involved in the system	**Existing Care Organization Models** Coordinated Care: minimum 2–3 professions working to coordinate care to deliver best practices (e.g. NCM, Pharmacist & Physician) Multi-disciplinary Care: 2 disciplines working together (e.g. Psych & GIM) Shared Care: specialty services (e.g. addiction psychiatry) lead the induction process & hands off to Internal Medicine/Primary Care to share longitudinal care Chronic Care: utilizing healthcare resources to self-empower individual management of chronic disease Physician Centric: single physician (or group of only physicians) working with available resources to manage OUD with BP/Methadone/NX	Implement Coordinated Care models with non-physician team members (i.e. RNs) to help manage patient appointments and lab results Evaluate effectiveness of multidisciplinary teams in providing comprehensive behavioral counseling and better outcomes Determine appropriate skillset needed by non-physician team members to appropriately delegate tasks for high quality care
*PERSON/TASKS*	**Person**: all of the individuals, both healthcare workers and patients, involved in the design of the work system **Tasks**: clinical processes and responsibilities of both the healthcare workers involved in the system as well as responsibilities for the patient (i.e. receiving medication, counseling attendance, etc.)	Large variation in type of skilled professionals providing support (e.g. nurses, pharmacists, counselors) Pharmacists roles and tasks (i.e. supervising dispensing, clinical appointments, management) dependent upon intervention Behavioral health providers ranged in training (i.e. PhD psychologists, certified addiction counselors, social workers)	Capitalize on various providers’ skillsets to deliver high quality care Employ clinical pharmacists for complicated medication dosing and management Increase clinical support (i.e. nursing) responsibility in management of patients
*PROCESS*	**Process**: the flow of actions or steps taken to provide patient care (e.g. order of delivery for intake, induction, maintenance, and follow up)	Use of non-physician staff to conduct patient intakes decreased physician work load Home inductions allowed patient autonomy and less frequent initial appointments Limited studies evaluated behavioral counseling approaches compared to medical management	Understand which patients can safely undergo home inductions Streamline home induction process to decrease care utilization during induction time period Utilize non-physician team members to conduct patient intakes Develop technologies and systems providing after hour support for patient care, data collection, & feedback Promote PCP management of stabilized patients on maintenance medications within specialty addiction treatment programs
*TECHNOLOGY & TOOLS*	**Technology and Tools**: components of the system including various information technologies like electronic health records, human factors characteristics of technologies (i.e. usability), and other technologies incorporated	Only 10 studies explicitly noted using patient treatment agreement Most studies used some form of a urine drug screen to monitor adherence Only 3 studies used panel management structure to keep track of patient level data	Standardize important tools (i.e. toxicology screenings & management structures) to monitor patient and population level outcomes
*PATIENT/ PROVIDER OUTCOMES*	**Patient Outcomes**: participant perceptions of the care delivery model, retention rates in the intervention, and health outcomes for the participant **Provider Outcomes**: provider perceptions of the care delivery model and system	The most commonly measured patient outcomes were retention in intervention, self-reported abstinence, and abstinence via/urine toxicology screens Less than half of the studies collected outcomes regarding other common primary care based comorbidities Provider outcomes were only discussed in 10 included trials Provider outcomes did highlight the benefits of coordinated care models	Gather patient-centered outcomes including management of physical and mental comorbidities Collect outcomes related to social determinants, social support, and improvement in work/personal level functioning Collect provider outcomes regarding appropriate levels of training to provide care Develop and evaluate provider support systems to provide ongoing education and prevent provider burnout

#### Environment

Studies were conducted in the following countries: U.S. (24), U.K. (3), Australia (2), Canada (2), Austria (1), France (1), Ireland (1), and Italy (1). All studies occurred in primary care centers; however, ten studies compared specialty versus primary care settings [27–36]. Some of the studies (n = 14) were conducted in academic primary care centers [8, 10–12, 37–47] while others (n = 14) occurred in private practice settings [28, 29, 32, 35, 47–55]. Five studies were in community health centers with limited resources [12, 56–59].

#### Organization

All of the studies meeting inclusion criteria studied buprenorphine (n = 25) and/or methadone (n = 12) treatment in primary care settings. Due to large variability in terminology and reporting, the interventions were grouped into at least one of five care models (e.g. collaborative care vs. integrated care). The most common type was a coordinated care model which had at least two different types of healthcare professionals actively communicating and working together to share care responsibilities (e.g. nurse case manager or pharmacist plus physician). Of the 32 “coordinated care” models, twelve relied upon a nurse case manager or other skilled nursing staff to lead and provide logistical support to the PCP [8, 10, 32, 35, 38, 51, 58, 60–64]. Often the nurse received training to provide some behavioral counseling [38, 58, 60, 65]. Pharmacists also provided assistance to PCPs by supervising medication dosages [49, 51, 53, 66, 67]. Multi-disciplinary models consisted of two physician disciplines working closely together within the same clinic (e.g. addiction psychiatry and internal medicine). For example, one study specifically evaluated the benefit of adding in-clinic behavioral counseling to standard medical management provided by a general internist for patients receiving methadone for heroin use [61]. Shared care models had specialty services lead the medication induction process (the first week of MAT where the physician determines the dosing, timing and treatment goals of the medication) and then later “handed-off” patients to general internal PCPs [33, 45, 56, 58]. In Cunningham et al (2008), patient induction was initiated by the pharmacist before transfer to the physician’s care [56]. The chronic care model, utilized healthcare resources to increase patients’ self-efficacy in managing their chronic disease [57]. Only two studies met this criterion [10, 34]. For example, one study used this framework to design a home induction protocol to empower patients to self-administer medications [34]. Last, the physician-centric model had a single physician or group of physicians working together to provide patient-centered MAT without major structural support from other provider types or disciplines. In Doolittle & Becker (2011), the physician independently counseled and treated the patient [68].

Studies were assigned to models via the criteria outlined above and could be categorized more than once to best capture treatment delivery. Most included studies (n = 32) had coordinated care models with 32 studies falling into two or more care models (e.g. coordinated care and multi-disciplinary care) [8, 10–12, 28–30, 32, 33, 38, 43, 45, 48–51, 56–58, 60, 61, 63, 64, 66, 67, 69–75]. Only three physician-centric studies existed, predominantly in community health centers, where physicians independently provided MAT [34, 68, 76].

#### Person/Tasks

Thirty-one studies included non-physician providers (e.g. nurses, pharmacists, counselors) to carry out tasks. [8, 10, 11, 27–30, 32, 33, 38, 45, 49–51, 56–60, 62–67, 69–73, 75, 77] The level of training and specific tasks managed by each non-physician provider varied across interventions. First, multiple studies used nurses as liaisons in coordinating care between PCPs and behavioral specialists. [8, 10, 32, 51, 58, 63] This configuration improved performance processes and collaborative work[14]. Both licensed practicing nurses (LPN) and advanced practicing nurses (NPs) were used as program coordinators to lead the intervention while supporting both patients and staff [10, 38, 60]. For example, Lucas et al (2010) had LPNs oversee patient-physician scheduling and assist with induction. Other studies used nursing staff to not only provide care collaboration, but to lead patient visits [8, 10, 32, 38, 51, 60, 62, 64–66]. However, only physicians could prescribe medications. Second, pharmacist roles and tasks varied across interventions. Multiple studies had pharmacists supervise dispensing of buprenorphine or methadone [27–30, 33, 49, 51, 56, 57, 63, 67, 71, 75, 77, 78]. The majority of these were conducted in the Europe [27–30, 51]. One U.S. study used a clinical pharmacist to provide physician guidance regarding appropriate dosing/tapering strategies [75] and to lead most patient follow-up appointments. Third, heterogeneity in formal training among clinicians for providing addiction counseling emerged. Behavioral counseling providers ranged from PhD-trained psychologists [65, 70] to certified addiction counselors [8, 10, 11, 32, 51, 58, 73, 74] to nurses with brief training in addiction counseling [38, 50, 66].

#### Process

Process focused on the flow of patient care within organizational models. Seven studies had a non-physician (e.g. nurse case manager) perform an initial detailed intake that often consisted of a physical and mental health history, allowing PCPs to focus their time on medication management [10, 38, 45, 49, 60, 74, 75]. Following the intake visit, studies varied on how they handled medication induction. Twenty-nine studies supervised patient induction with frequent appointments and supervised medication dosing [8, 10, 11, 27–30, 32–34, 38, 49–51, 56–58, 60, 62, 63, 65, 67, 69–72, 75–77]. In contrast, four studies evaluated “home” inductions, which increased patient autonomy via a specific plan for how to first begin self-treatment with the chosen medication [55, 57, 68, 71]. Following induction, frequency of appointments with staff ranged from daily to quarter-annually depending upon patient needs and intervention design. Behavioral counseling appointments were often coordinated with medical management appointments [11, 62, 65, 69, 70, 72]. In cases requiring more intense addiction counseling/treatment, nineteen studies had a plan for referral to specialty services [8, 11, 12, 27, 30, 32, 37, 38, 50, 51, 56, 62, 63, 70, 72–75, 79].

Only four studies formally tested if counseling modality or duration affected treatment outcomes [9, 43, 80, 81]. Of these four studies, only two demonstrated that their form of counseling was more efficacious (e.g. patients undergoing CBT had higher rates of negative urine toxicology screens or >80% Quality Health Indicator score) than medical management alone possibly because of their adaptive, stepped-care treatments or highly integrated care teams with numerous support staff [9, 81].

#### Technology/Tools

Technology/Tools focused on electronic and non-electronic tools that helped manage data and monitor patient outcomes like patient agreements, drug screenings, and electronic information technology systems. Ten studies had formal treatment agreements (“contract”) between patients and providers to outline consequences for continued drug misuse. [8, 11, 37, 38, 51, 68, 69, 75, 77] Most interventions (n = 29) noted that they used urine drug screening as a tool to monitor adherence to medication and drug misuse; although, there was no standardization in what drugs were screened or how often.

Regarding technologies, three studies noted that they had a panel management structure to monitor patient level data (i.e. urine toxicology screens, drug tests, etc.). [10, 75, 81] Four studies noted using electronic medical records to facilitate treatment team member communication and to document patient updates [58, 63, 72, 75]. No studies utilized home-based or web-based counseling modalities [73].

#### Patient outcomes

Reported patient outcomes varied. However, most studies (n = 25) reported patient-level retention within treatment. At 3 months, nineteen interventions achieved at least 60% retention. Some studies evaluated if patients had self-reported abstinence (n = 15) while others incorporated quantitative measures of abstinence (i.e. urine toxicology screens; n = 22).

Few studies asked about patient perceptions of the care delivery models. One study evaluating a coordinated care model for patients receiving MAT obtained patient feedback regarding care with 90% of patients reporting overall satisfaction with the care model.[52] Additionally, less than half of the interventions (n = 11) assessed management of other common primary-care comorbidities and age-appropriate screenings [10, 12, 38, 45, 49, 50, 57, 58, 60, 64, 68]. One of these studies evaluated what percentage of patients were meeting nine quality health indicators (QHI) of an age appropriate health screening per CDC criteria via retrospective chart review [58]. Greater than 3 months of treatment on buprenorphine was positively associated with achieving a recommended QHI screening score [(AOR) = 2.19; 95% confidence interval (CI) = 1.18–4.04)]. Similarly, Roll et al (2015) surveyed 28 patients receiving shared medical appointments for buprenorphine management therapy. They found that 60% of patients reported learning more about comorbidities like Hepatitis C and 43% reported receiving appropriate immunizations since starting the intervention. [54] The BHIVES collaborative was a ten site intervention evaluating the use of buprenorphine/naloxone for patients with both HIV and OUD. The evaluation used mixed-methods and reported patient outcomes on OUD treatment, HIV treatment, HIV related quality of life, patient perspectives on the intervention, and overall quality of life. [12, 22, 23, 25–27, 82].

#### Provider outcomes

Provider level outcomes were only reported in ten of the included studies [28, 30, 32, 49, 63, 69, 72, 74, 77, 83]. Of these, six studies asked providers qualitatively about barriers and facilitators of the intervention’s success [12, 32, 49, 50, 69, 74].

Themes that emerged from provider outcome data included provider education, cost-related barriers, and benefits of a coordinated-care approach. Three studies noted that providers felt under-trained or that some training in providing the chosen medication (i.e. buprenorphine or methadone) or substance abuse treatment was important. O’Connor et al (1998) found that the intervention itself increased provider confidence in treating patients with OUD. [32, 50, 69, 77]. However, Fiellin et al (2004) reported that physicians felt “adequately prepared for much of the care they provided,” but requested that further training be offered with respect to medication tapering, billing, and additional training for support staff [52]. Nine studies reported that providers felt that there were benefits of coordinated care [8, 38, 49, 51, 58, 60, 75, 77, 81]. In Drainoni et al (2014), providers noted that the RN/Counselor taking ownership of the program was pivotal to program success[8]. Likewise, in Weiss et al (2011), providers noted that coordinated care is crucial in a busy academic setting where physicians had limited availability during clinic hours [12].

#### Presence of SEIPS domains in good quality studies with high patient retention

**Fig 2:**
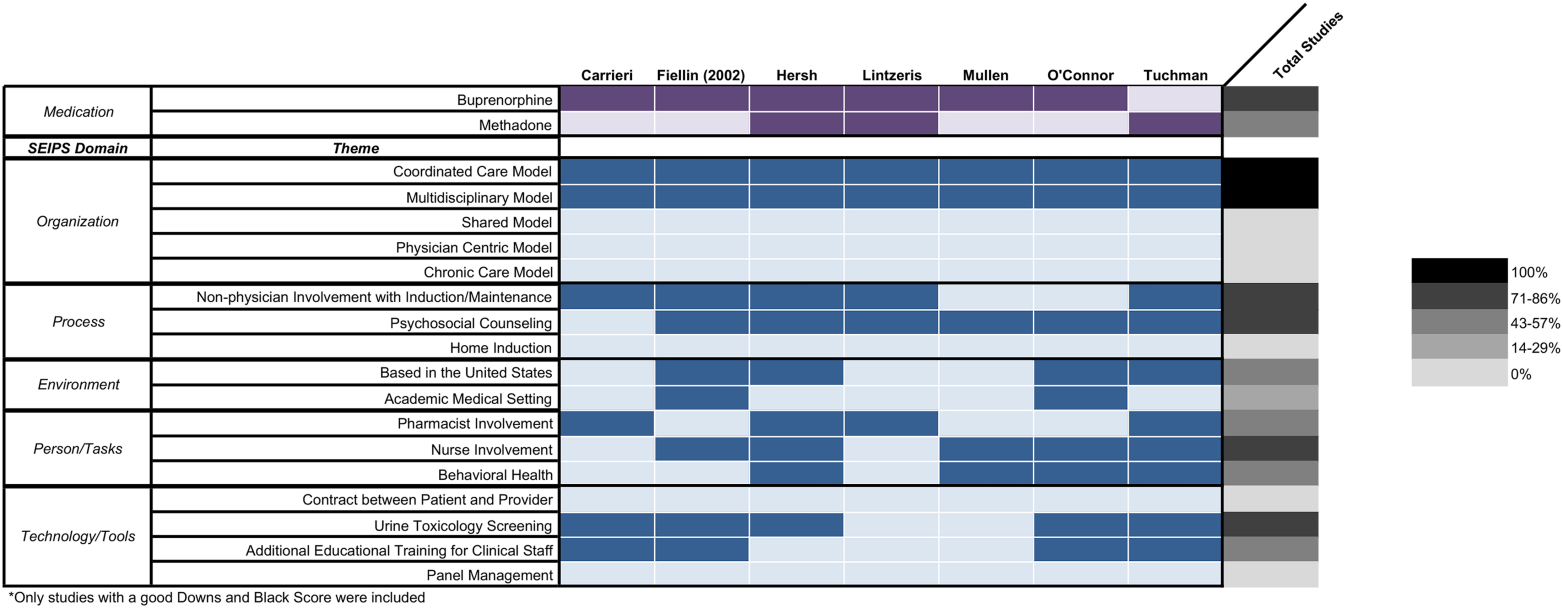
Presence of SEIPS domains in good quality studies with high patient retention*.

There was heterogeneity in study caliber, medications and dosages, reported patient outcomes, and duration of study making it difficult to perform any meta-analysis with the 35 included studies. However, in order to describe which SEIPS domains may be associated with successful treatment and establish a level of standardization, we defined successful studies as interventions that achieved 60% retention rates at 3 months and received a good score with our validated risk assessment tool. Seven studies met this metric for success (Fig 2) [27, 31, 32, 42, 52, 84, 85].

All seven successful studies used coordinated care models with multidisciplinary teams. Six of the studies used buprenorphine as the medication, had a modality for delivering behavioral counseling (although not necessarily through trained behavioral health specialists), used nurses as part of the care team, and monitored treatment outcomes with urine toxicology screens. With respect to technology/tools none of the included studies used patient and provider contracts or panel management structures. In addition, only 4/7 included studies provided additional educational training for the clinical staff.

### Risk of bias

No study scored an excellent quality/risk of bias score: sixteen interventions scored good, seventeen scored fair, and two were poor for their reporting of patient outcomes significance (i.e. power within the study to detect differences in outcomes) and their lack of both external and internal validity[51, 54].

## Discussion

Few comparisons of primary care models for OUD exist. Our study addresses this gap in knowledge by using the SEIPS domains to categorize features of MAT interventions to better understand specific structural, process and outcome elements of primary care models. The range of studies spanned small physician-led interventions within single clinics to large RCTs with multi-disciplinary teams in academic settings. Based on our synthesis of peer-reviewed literature, we report the current structures and processes of primary care-based OUD MAT models and present a proposed research agenda for future studies within each SEIPS domain.

Coordinated care models (with non-physician team members such as RNs helping manage patient appointments and lab results) are by far the most common delivery structures studied. There was some indication that physicians felt this program model allowed for improved team communication and higher quality of care delivery [8, 12, 47, 51, 52, 66, 86]. Similarly, multidisciplinary teams can promote comprehensive behavioral health counseling in addition to standard PCP-led counseling during routine primary care appointments. However, future studies will need to further delineate the cost and feasibility of these resources in settings where multidisciplinary care is inaccessible or not viable. Ideally, studies would evaluate a physician-centered model against models with varying degrees of care coordination with randomized controlled trials but such studies are costly. With the need to rapidly disseminate primary care based models to provide MAT, this study highlights that policy makers and health care professionals should strive to provide and pragmatically evaluate at the very least, the provision of some coordinated care. These models may include smaller teams or clinical partnerships (i.e. physician-RN teams, physician-pharmacist teams) that are more common across resource settings.

The effective use of clinicians’ skillsets can improve overall care delivery. For example, clinical pharmacists can provide medication dosing and management rather than only supervised medication-taking. In terms of behavioral counseling, more research is needed to identify the optimal level of training necessary for OUD care delivery. Studies have suggested that additional counseling beyond the scope of the physician is ineffectual for certain outcomes [43, 87]. However, future research will need to understand whether certain patient populations benefit more or less from additional counseling to help allocate limited behavioral health resources to those patients that would derive the most benefit.

Home inductions proved successful (≥ 60% retention) for select patients, but future research is still needed to recommend the routine use of home inductions [55, 57, 71] and to identify the patient characteristics that are associated with successful and non-successful home inductions. Furthermore, the use of RNs or other support staff to conduct patient intakes (i.e. physicals, mental health screenings) helped disencumber physician responsibilities. [8, 10, 11, 32, 38, 50, 51, 58, 60, 62–64, 78] Providing patients with “after hours” support was another component of care noted in numerous studies. [39, 45, 55–57, 60, 71, 74] More research is needed to assess the effects of augmenting such support with the use of mobile technology (i.e. telehealth, text messages, emails) to improve the process of providing 24-hour support. Only three studies examined the influence of addiction specialists transferring stabilized patients to primary care [30, 33, 63]. This approach may appeal to primary care workforces wanting to expand access to MAT through a stepped-care approach (i.e. providing stabilized patients maintenance dosing and managing comorbidities), but who are less comfortable providing initial MAT induction.

Wide variation in the use of toxicology screens, patient contracts, and data management structures existed and were largely underdeveloped. Our conclusions correlated with findings from a systematic review examining the use of urine drug screens and treatment agreements in patients with chronic pain, and found that more research is needed to standardize these tools to not only monitor patient level outcomes, but provide population-level feedback to care teams [88]. Additionally, much of the technological aids discussed were relatively nascent with Mullen et al (2012) noting that a more sophisticated data management structure would be helpful [31]. There was substantial heterogeneity with respect to patient and provider outcomes measured. Patient retention was the most common outcome reported, yet there was no uniform definition given dissimilar program lengths between studies (i.e. 1 month to 2 years). Additionally, there was a lack of consideration given to the remitting nature of OUD. Many of the study samples excluded co-dependence on other illicit substances like benzodiazepines, leading to possible confounders that could affect the analyses.

Few studies evaluated provider outcomes such as quality of medical care, physician perceptions, and factors related to care delivery in relation to a primary care-based MAT intervention. While other studies in the literature measure provider barriers to delivering OUD treatment in primary care settings [89–91], these studies do not measure provider outcomes concurrently to testing patient level outcomes within specified care model structures. This gap indicates key areas for future research such as determining the appropriate level of provider training, testing provider training and mentoring support mechanisms such as the Providers' Clinical Support System for Medication Assisted Treatment [92] and Project ECHO (Extension for Community Health Outcomes) a video-based distance education program, and determining how to prevent physician burnout. [85]

Our study could not make any definitive statements regarding whether particular care models or treatment elements are strongly associated with favorable patient outcomes compared to alternatives. However, when we did look across studies with good quality and high patient retention there was a pattern suggesting that successful studies used coordinated, multidisciplinary models to support physicians in delivering MAT. The majority of the seven studies did not use tools such as patient/provider contracts nor did they provide additional clinical staff training suggesting that they may not be necessary to successfully carry out primary care based MAT.

Our study has limitations. First, because of both the heterogeneity in reporting of outcomes and variability in medications used and dosages, we were unable to perform a pooled meta-analysis to clearly link structural domains identified via SEIPS to health outcomes. Second, not all of the studies included had randomized designs. Potential for bias and confounding should be considered, though it was formally assessed within our methods. Third, we were unable to quantitatively evaluate which particular SEIPS domains contributed to intervention success or failure. Fourth, we only included studies that were published in peer-reviewed literature. Therefore, we did not capture interventions that may be in the pilot phase or have outcomes presented via other “grey” literature such as websites/forums.

Our study has several strengths. To our knowledge, this is the first systematic review that describes both patient outcomes and structural organization of interventions to treat OUD in primary care settings both domestically and internationally. Second, by using the SEIPS framework, we described systems design elements within each intervention rather than focusing only on the broad organizational framework of the intervention. Not all primary care settings have access to the same resources with some unable to structurally accommodate a multi-person coordinated care team. However, we provide details on how to improve systems (e.g. person, tasks, and process) depending on the resources that are available in various settings.

There is variability in regulations for treatment programs, payment models, and provider training structures which may limit or enhance the ability of health systems to provide high quality multidisciplinary, coordinated care with the most cost-effective, efficacious OUD interventions. As the US allocates funding towards expanding MAT access, programs receiving such funding would benefit from considering the intervention models found to support MAT implementation in prior studies [93]. By evaluating not only patient efficacy, but also structural characteristics of primary care models for delivering MAT, this review provides key insights for PCPs and researchers about ways to build upon existing resources and personnel to more effectively deliver OUD treatment. Specifically, this study identified key components of primary care OUD delivery models. As we continue to grapple with the global rise of opioid-related morbidity and mortality, this review can help enhance the rapid dissemination of effective OUD treatment programs across diverse settings.

## Supporting information

S1 Table Search terms. (TIFF)

S2 Table Inclusion and exclusion criteria. (TIFF)

S1 Fig PRISMA checklist. (TIFF)
