# Supplementary figures and images for: Primary care models for treating opioid use disorders: What actually works? A systematic review
Source: PLoS One. 2017 Oct 17;12(10):e0186315. doi: 10.1371/journal.pone.0186315 (PMC5645096; doi:10.1371/journal.pone.0186315)

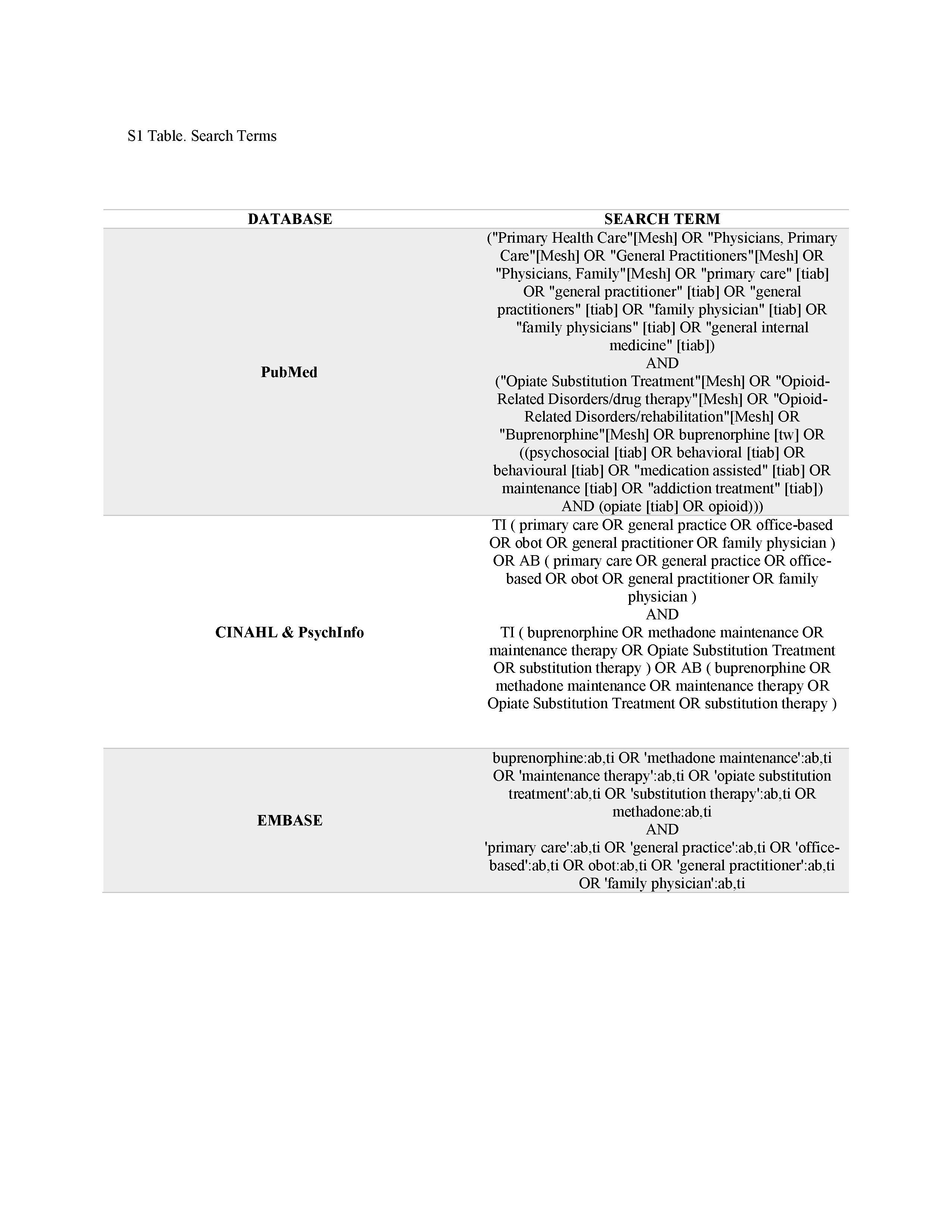

Supplement: S1 Table — (TIFF) [file pone.0186315.s001.tiff]

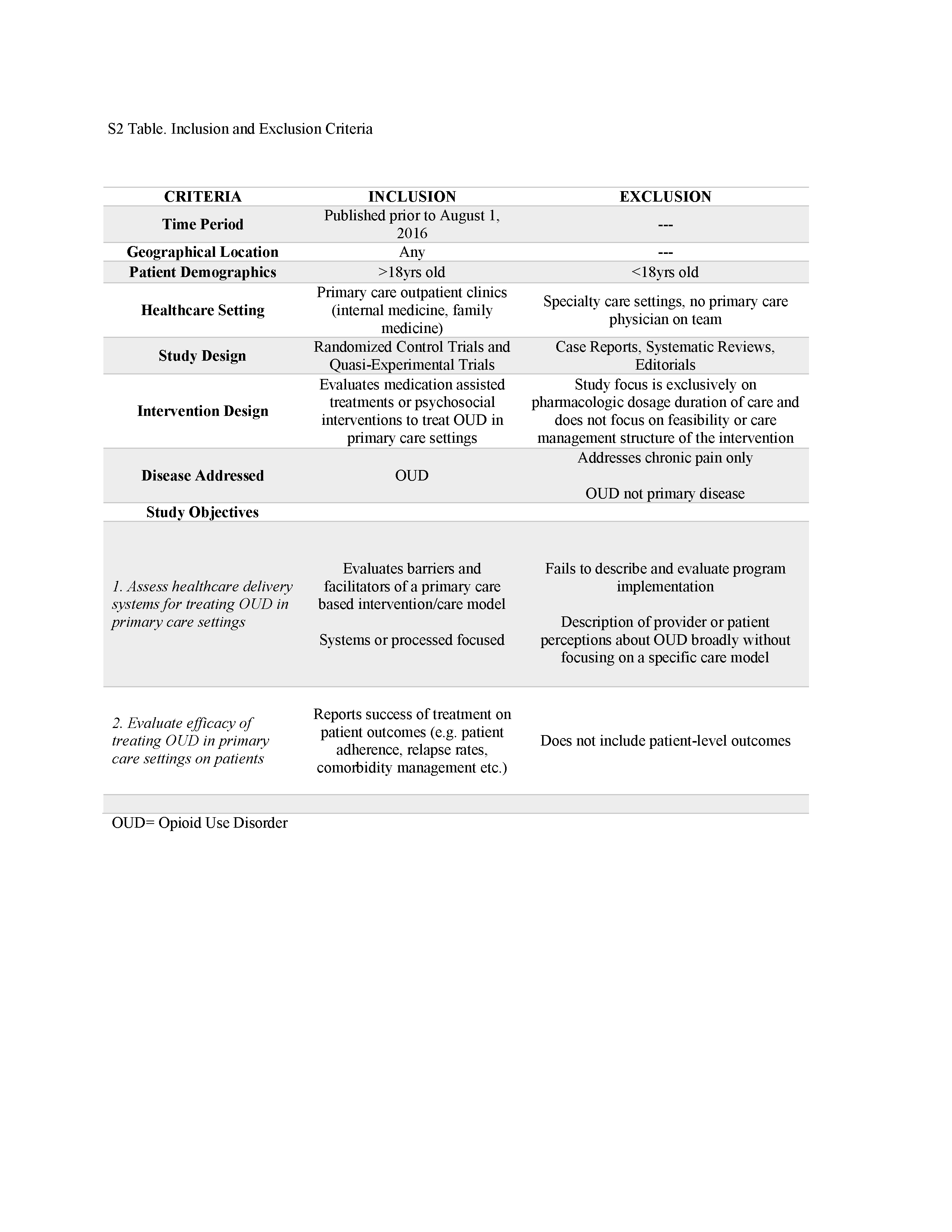

Supplement: S2 Table — (TIFF) [file pone.0186315.s002.tiff]

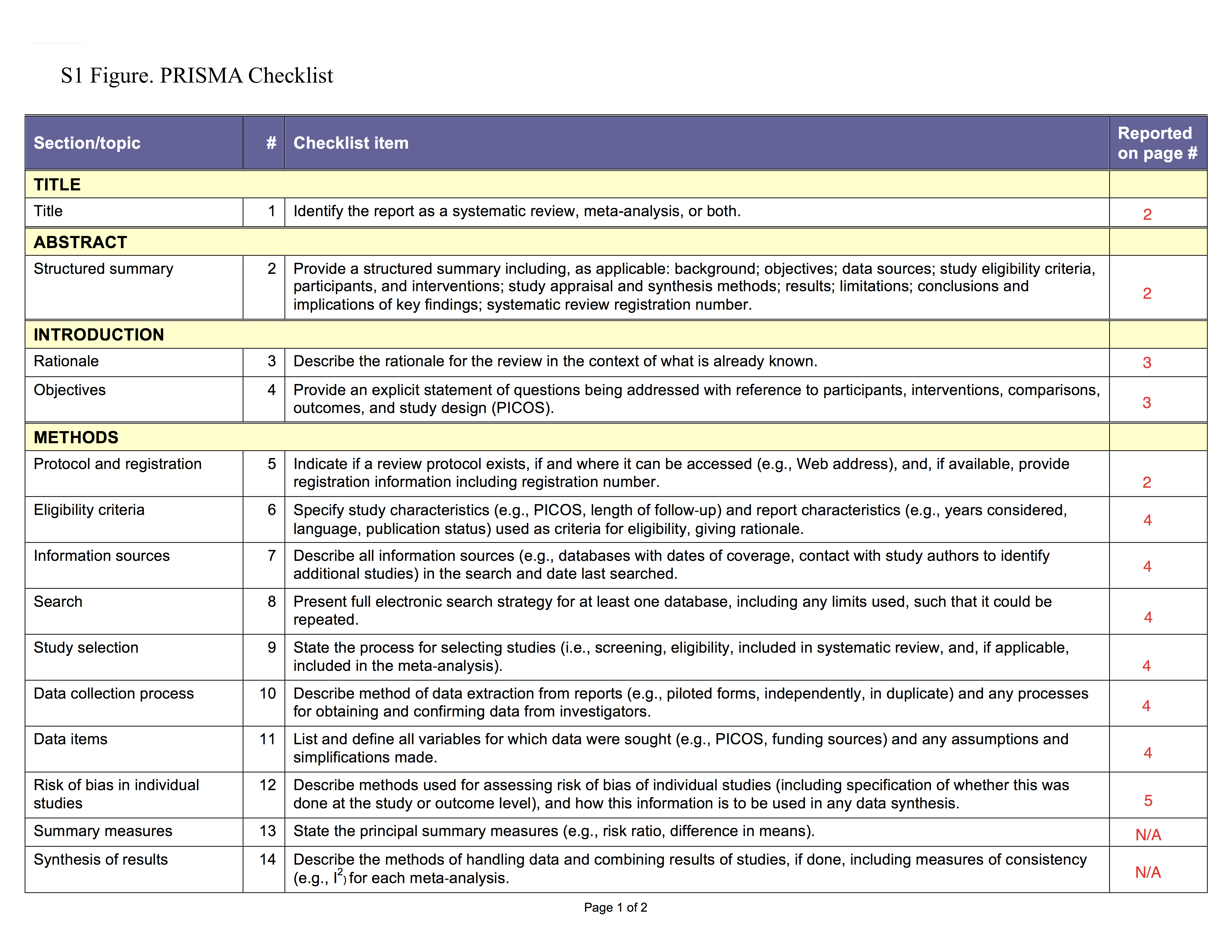

Supplement: S1 Fig — (TIFF) [file pone.0186315.s003.tiff]
